# Regioselective alkynylation followed by Suzuki coupling of 2,4-dichloroquinoline: Synthesis of 2-alkynyl-4-arylquinolines

**DOI:** 10.3762/bjoc.5.32

**Published:** 2009-07-01

**Authors:** Ellanki Amarender Reddy, Aminul Islam, K Mukkanti, Venkanna Bandameedi, Dipal Ranjan Bhowmik, Manojit Pal

**Affiliations:** 1 Dr. Reddy’s Laboratories Limited, Bollaram Road, Miyapur, Hyderabad 500049, Andhra Pradesh, India; 2 Chemistry Division, Institute of Science and Technology, JNT University, Kukutpally, Hyderabad 500072, Andhra Pradesh, India; 3 New Drug Discovery, R&D Center, Matrix Laboratories Ltd., Anrich Industrial Estate, Bollaram, Jinnaram Mandal, Medak District, Andra Pradesh 502 325, India (present address: Institute of Life Science, University of Hyderabad Campus, Gachibowli, Hyderabad 500 046, Andhra Pradesh, India)

**Keywords:** alkyne, boronic acid, catalysis, 2,4-dichloroquinoline, palladium, water

## Abstract

A two step synthesis of 2-alkynyl-4-arylquinolines has been accomplished via Pd/C-mediated regioselective C-2 alkynylation of 2,4-dichloroquinoline in water followed by Suzuki coupling at C-4 of the resulting 4-chloro derivative.

## Introduction

**Figure 1:**
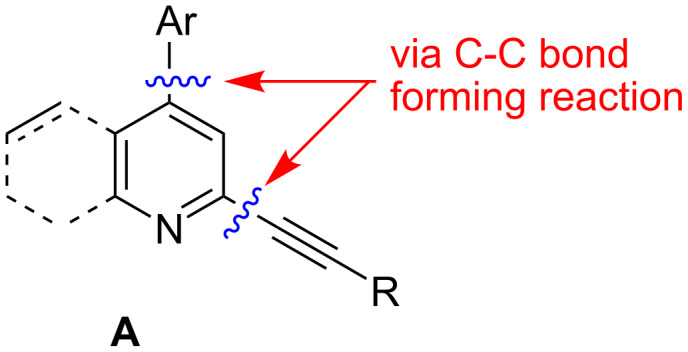
2-Alkynyl-4-aryl pyridine and its benzo derivative.

2-Alkynyl pyridine and its benzo (i.e. quinoline) derivative possessing an aryl group at the C-4 position (**A**, Figure 1) have attracted considerable interest due to their utility in the development of compounds of potential pharmacological interest [1–3].

**Scheme 1:**
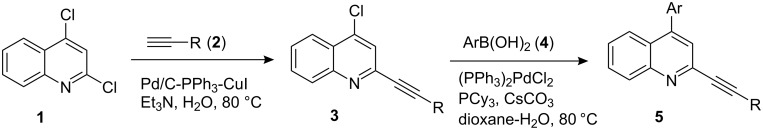
Sequential synthesis of 2-alkynyl-4-arylquinolines from 2,4-dichloroquinoline under palladium catalysis.

2-Alkenyl/alkynylquinolines, have been reported to possess anti-retroviral properties [4]. Only few methods are known for the synthesis of **A**. Considering the possible C–C bond forming reactions on a pyridine/quinoline ring (Figure 1), the synthesis of **A** can be carried out following two main strategies e.g. (a) arylation at C-4 followed by alkynylation at C-2 or (b) alkynylation at C-2 followed by arylation at C-4. Methodologies based on strategy ‘a’ have been reported earlier. For example, Sonogashira coupling of a terminal alkyne with 2-chloro-4-aryl substituted quinoline [3] in the presence of (PPh_3_)_2_PdCl_2_-CuI or treatment of 4-aryl pyridine-*N*-oxide with alkynyl Grignard [5] provided the required quinoline or pyridine derivatives, respectively. Notably, synthesis of **A** following the strategy ‘b’ has not been explored. In our effort towards the synthesis of quinoline derivatives of potential biological significance we have reported Pd/C-mediated regioselective C-2 alkynylation of 2,4-dichloroquinoline in water [6]. However, only one example of regioselective C-2 alkynylation was reported and no detailed study has been carried out previously. Herein we report the preparation of a series of 2-alkynyl-4-chloroquinoline (**3**) followed by successful Suzuki coupling at C-4 of compound **3** leading to the corresponding 4-arylated derivatives (**5**) in good yields (Scheme 1). To the best of our knowledge this is the first synthesis of 2-alkynyl-4-arylquinolines following such a strategy.

## Results and Discussion

A number of 2-alkynyl-4-chloroquinolines (**3**) were prepared via coupling of 2,4-dichloroquinoline (**1**) in the presence of 10% Pd/C (10 mol%), PPh_3_ (20 mol%) and CuI (5 mol%) as a catalyst system in water. The results are presented in Table 1. Both aryl and alkyl substituted terminal alkynes participated well in this C–C bond forming reaction to afford the desired product in good yields. The reaction was found to be highly selective for mono-substituted product and no dialkynylated product was isolated from the reaction mixture. Moreover, the reaction displayed good regioselectivity for C-2 alkynylation though the formation of C-4 alkynylated product cannot be ruled out completely. Regioselectivity for C-2 alkynylation was confirmed by NOE (Nuclear Overhauser Effect) studies using compound **3a**. Irradiation of protons of the benzene ring attached to the alkynyl group resulted in enhancement of the singlet at δ 8.03 assigned to the C-3 hydrogen of the quinoline ring. If the alkyne was at C-4, NOE enhancement at C-5 is expected in addition to C-3. It is note worthy that the use of the Sonogashira coupling or its modified form has been employed for the preparation of 2-alkynylquinolines or related derivatives earlier [7–17].

**Table 1:** Pd/C-mediated [Pd/C (10 mol%)–CuI (5 mol%)–PPh_3_ (20 mol%)] synthesis of 2-alkynyl-4-chloroquinolines (**3**).^a^

Entry	Terminal alkynes (**2**)	Time (h)	Products (**3**)^b^	Yield (%)^c^

1.	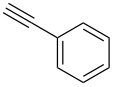 **2a**	10	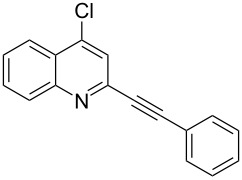 **3a**	88
2.	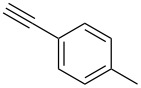 **2b**	8	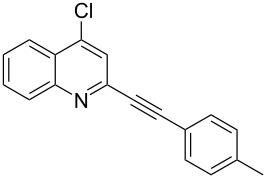 **3b**	85
3.	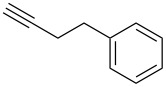 **2c**	10	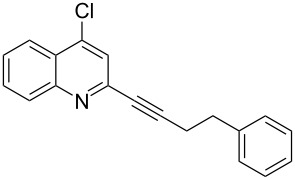 **3c**	90
4.	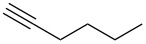 **2d**	12	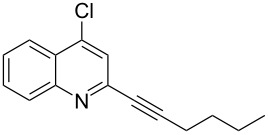 **3d**	92
5.	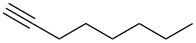 **2e**	12	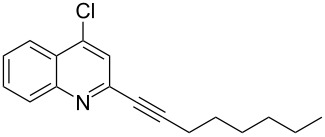 **3e**	90
6.	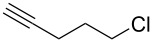 **2f**	8	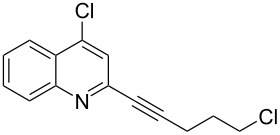 **3f**	87

^a^All the reactions were carried out by using compound **1** (1.0 equiv), terminal alkyne **2** (1.5 equiv), 10% Pd/C (0.026 equiv), PPh_3_ (0.20 equiv), CuI (0.05 equiv), and Et_3_N (3.0 equiv) at 80 °C. ^b^Identified by ^1^H NMR, IR, and MS. ^c^Isolated yields.

Next, in order to prepare 2-alkynyl-4-arylquinolines (**5**) we planned to exploit the reactivity of chloro group of **3** towards Suzuki arylation reaction. Accordingly, a variety of arylboronic acids were coupled with **3** and results of this study are summarized in Table 2. The Suzuki reaction was carried out using arylboronic acids in the presence of (PPh_3_)_2_PdCl_2_ as a catalyst, CsCO_3_ as a base, tricyclohexyl phophine, (PCy_3_) as a ligand in dioxane–water at 80 °C. The arylboronic acids used in this reaction include phenylboronic acid (entries 1–6, Table 2), 3-methoxyphenylboronic acid (entry 7, Table 2) and 4-fluorophenyl boronic acid (entry 8, Table 2), all of which participated well in the coupling reaction with **3**. A number of 2-alkynyl-4-arylquinolines (**5**) were prepared in good to excellent yields without affecting the alkynyl substituents present in compound **3**.

**Table 2:** Synthesis of 2-alkynyl-4-arylquinolines (**5**).

Entry	4-Chloro compd (**3**)	Arylboronic acid (**4**)	Product^a^ (**5**)	Time (h)	Yield (%)^b^

1.	**3e**	phenylboronic acid (**4a**)	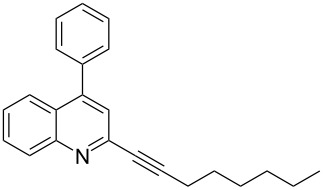 **5a**	4	83
2.	**3b**	**4a**	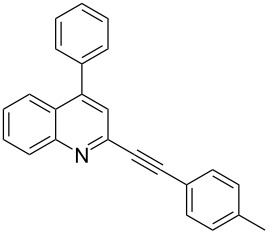 **5b**	3	88
3.	**3c**	**4a**	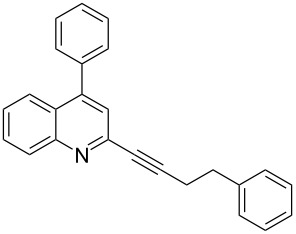 **5c**	2	87
4.	**3d**	**4a**	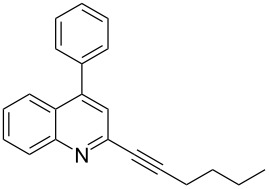 **5d**	4	82
5.	**3a**	**4a**	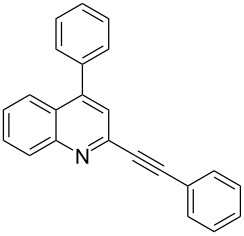 **5e**	2	84
6.	**3f**	**4a**	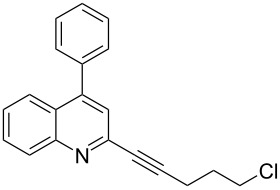 **5f**	3	79
7.	**3a**	3-methoxy-phenylboronic acid (**4b**)	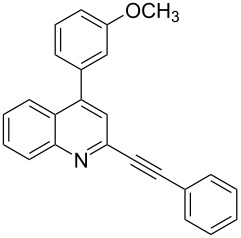 **5g**	2	86
8.	**3a**	4-fluoro-phenylboronic acid (**4c**)	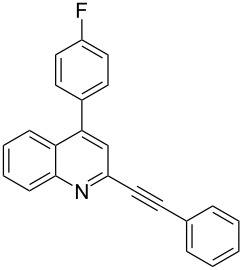 **5h**	2	86

^a^Identified by ^1^H NMR, IR, and MS. ^b^Isolated yields.

**Scheme 2:**
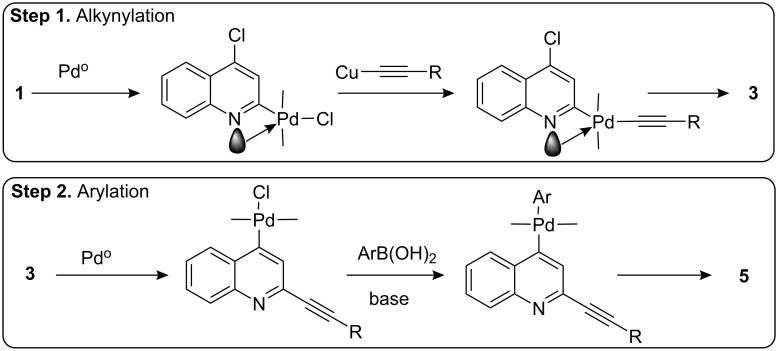
The reaction mechanism of stepwise C–C bond forming reactions.

The reaction mechanism of the present stepwise C–C bond forming reactions consisting of alkynylation followed by arylation is shown in Scheme 2. The Pd/C–Cu mediated coupling of 2,4 dichloroquinoline (**1**) with terminal alkynes (**2**) in water proceeds via normal Sonogashira pathway [6]. Due to the presence of electronegative nitrogen atom the chloro group at the azomethine carbon is more susceptible to undergo oxidative addition with Pd(0) than chloro group at C-4. Moreover, the coordination of quinoline nitrogen to the palladium [18–19] controls the regioselectivity in alkynylation of 2,4-dichloroquinoline at C-2 position. The 2-alkynyl-4-chloroquinolines **3** thus formed then undergo Suzuki reaction in the next step. Oxidative addition of Pd^0^ generated in situ to compound **3** followed by trans-organometallation of the resultant aryl-palladium complex formed with arylboronic acids provides the desired compound **5**.

## Conclusions

In conclusion, a two-step method consisting of alkynylation followed by arylation has been developed for the synthesis of 2-alkynyl-4-arylquinolines. The alkynylation step involved Pd/C–Cu mediated regioselective C-2 alkynylation of 2,4-dichloroquinoline in water to afford 2-alkynyl-4-chloroquinoline. The arylation step is a Pd-mediated (Suzuki) coupling of 2-alkynyl-4-chloro derivative with arylboronic acids in aqueous media to give the target compounds. The process is amenable to the diversity-oriented synthesis of quinoline derivatives of potential pharmacological significance and may therefore find wide usage in organic/medicinal chemistry.

## Experimental

General Procedure for the preparation of compound **5**: A mixture of alkyne **3** (1.0 mmol) and (PPh_3_)_2_PdCl_2_ (0.05 mmol) in dioxane (5.0 mL) was stirred for 10 min under nitrogen at room temperature and then heated to 80 °C. To this mixture was added a solution of PCy_3_ (0.05 mmol) and CsCO_3_ (3.5 mmol) dissolved in water (3.0 mL) and arylboronic acid (1.5 mmol) dissolved in dioxane (3.0 mL) at the same temperature. The mixture was stirred at 80 °C according to the time indicated in Table 2. After completion of the reaction the mixture was cooled to room temperature, concentrated under vacuum and the residue was extracted with EtOAc (3 × 30 mL). The organic layers were collected, combined, washed with cold water (3 × 30 mL), dried over anhydrous Na_2_SO_4_ and concentrated under vacuum. The crude product was purified by column chromatography on silica gel, using light petroleum ether (60–80 °C)-ethyl acetate to afford the desired product. Spectral data for selected compounds; Compound** 5a**; light brown gum, Rf (20% ethyl acetate/n-hexane) 0.21; ^1^H NMR (CDCl_3_, 400 MHz) δ 8.14 (d, *J* = 8.0 Hz, 1H), 7.85 (d, *J* = 8.0 Hz, 1H), 7.69 (t, *J* = 7.8 Hz, 1H), 7.50–7.41 (m, 7H), 2.49 (t, *J* = 7.0 Hz, 2H), 1.69–1.21 (m, 8H), 0.91–0.8 (m, 3H); IR (cm^−1^, neat) 2927, 2225, 1587, 1543, 1357; m/z (ES Mass) 314 (M+1, 100%); ^13^C NMR (CDCl_3_, 50 MHz) 148.5, 143.6, 137.5 (2C), 129.6 (3C), 128.4 (2C), 126.7 (2C), 125.5 (3C), 124.3, 92.2, 81.0, 76.3, 31.3, 29.6, 28.0, 24.6, 22.5; HRMS (ESI): calcd for C_23_H_23_N (M+H)^+^ 314.1909, found 314.1896. Compound **5b**, low melting solid, R_f_ (20% ethyl acetate/n-hexane) 0.28; ^1^H NMR (CDCl_3_, 400 MHz) δ 8.20 (d, *J* = 7.8 Hz, 1H), 7.87 (d, *J* = 7.8 Hz, 1H), 7.75–7.71 (m, 1H), 7.56–7.47 (m, 9H), 7.25–7.17 (m, 2H), 2.38 (s, 3H); IR (cm^−1^, Neat) 2924, 2216, 1583, 1541; m/z (ES Mass) 320 (M+1, 100%); ^13^C NMR (CDCl_3_, 50 MHz) 148.6, 148.5, 143.2 (2C), 139.3 (2C), 137.3 (2C), 134, 132, 129.5 (3C), 129.3 (2C), 126.9 (2C), 125.6 (2C), 124.4, 118.9, 90.2, 88.8, 29.0; HRMS (ESI): calcd for C_24_H_17_N (M+H)^+^ 320.1439, found 320.1454.

## Supporting Information

File 1 Spectral data of 2-alkynyl-4-arylquinolines **5c**–**h**.
