# Supplementary material for: Regioselective alkynylation followed by Suzuki coupling of 2,4-dichloroquinoline: Synthesis of 2-alkynyl-4-arylquinolines
Source: Beilstein J Org Chem. 2009 Jul 1;5:32. doi: 10.3762/bjoc.5.32 (PMC2748691; doi:10.3762/bjoc.5.32)
Supplement: File 1 — Spectral data of 2-alkynyl-4-arylquinolines 5c–h. [file Beilstein_J_Org_Chem-05-32-s001.doc]

Supporting Information

**Regioselective alkynylation followed by Suzuki coupling of 2,4-dichloroquinoline: Synthesis of 2-alkynyl-4-arylquinolines**

Ellanki Amarender Reddy^1,2^, Aminul Islam^1^, K. Mukkanti^2^, Venkanna Bandameedi^1^, Dipal Ranjan Bhowmik^1^ and Manojit Pal*^,3^

Address:

^1^Dr. Reddy.s Laboratories Limited, Bollaram Road, Miyapur, Hyderabad 500049, Andhra Pradesh, India; ^2^Chemistry Division, Institute of Science and Technology, JNT University, Kukutpally, Hyderabad 500072, Andhra Pradesh, India and ^3^New Drug Discovery, R&D Center, Matrix Laboratories Ltd., Anrich Industrial Estate, Bollaram, Jinnaram Mandal, Medak District, Andra Pradesh 502 325, India (present address: Institute of Life Science, University of Hyderabad Campus, Gachibowli, Hyderabad 500 046, Andhra Pradesh, India).

E-mail: Manojit Pal- manojitpal@rediffmail.com

* Corresponding author

**Spectral data of 2-alkynyl-4-arylquinolines (5c−h)**

Compound **5c**; low melting solid, Rf (20% ethyl acetate/n-hexane) 0.18; ^1^H NMR (CDCl_3_, 400 MHz) δ 8.14 (d, *J* = 8.2 Hz, 1H), 7.86 (d, *J* = 8.3 Hz, 1H), 7.73–7.69 (m, 2H), 7.55–7.45 (m, 5H), 7.39–7.21 (m, 6H), 3.01 (t, *J* = 7.5 Hz, 2H), 2.19 (t, *J* = 7.5 Hz, 2H); IR (cm^−1^, neat) 2924, 2227, 1587, 1452; m/z (ES Mass) 334 (M+1, 100%); ^13^C NMR (CDCl_3_, 200 MHz) 148.1, 147.7, 144.2, 140.2, 138.1, 129.0 (5C), 128.4 (3C), 127.6 (3C), 126.0 (2C), 125.7 (2C), 122.0, 90.5, 88.7, 35.0, 23.0; HRMS (ESI): calcd for C_25_H_19_N (M+H)^+^ 334.1596; found 334.1582.

Compound **5d**, yellow liquid, Rf (20% ethyl acetate/n-hexane) 0.25; ^1^H NMR (CDCl_3_, 400 MHz) δ 8.14 (d, *J* = 8.3 Hz, 1H), 7.86 (d, *J* = 8.3 Hz, 1H), 7.71 (t, *J* = 6.8 Hz, 1H), 7.54–7.41 (m, 7H), 2.5 (t, *J* = 7.2 Hz, 2H), 1.66–1.80 (m, 4H), 0.95 (m, 3H); IR (cm^−1^, neat) 2929, 2223, 1587, 1541, 1355; m/z (ES Mass) 286.2 (M+1, 100%); ^13^C NMR (CDCl_3_, 200 MHz) 148.4, (2C), 143.5, 137.3, 129.5 (3C), 128.4 (2C), 126.6 (2C), 125.4 (3C), 124.2, 91.0, 81.0, 76.0, 30.3, 22.0, 19.1; HRMS (ESI): calcd for C_21_H_19_N (M+H)^+^ 286.1596; found 286.1593.

Compound **5e,** pale yellow solid, Rf (20% ethyl acetate/n-hexane) 0.22; ^1^HNMR (CDCl_3_, 400 MHz) δ 8.20 (d, *J* = 8.7 Hz, 1H), 7.88 (d, *J* = 8.7 Hz, 1H), 7.75–7.71 (m, 1H), 7.67–7.64 (m, 2H), 7.58–7.44 (m, 8H), 7.39–7.35 (m, 2H); IR (cm^−1^, neat) 2926, 2214, 1585, 1492, 1359; m/z (ES Mass) 306.1 (M+1, 100%); ^13^C NMR (CDCl_3_, 200 MHz) 148.6 (2C), 143.3 (2C), 139.3 (2C), 137.4 (2C), 132.0 (2C), 129.7 (6C), 128.5 (2C), 125.4 (2C), 124.3, 90.2, 88.9; HRMS (ESI): calcd for C_23_H_15_N (M+H)^+^ 306.1283; found 306.1283.

Compound **5f**; brown liquid, Rf (30% ethyl acetate/n-hexane) 0.27; ^1^H NMR (CDCl_3_, 400 MHz) δ 8.14 (d, *J* = 8.3 Hz, 1H), 7.86 (d, *J* = 8.3 Hz, 1H), 7.71 (t, *J* = 6.8 Hz, 1H), 7.54–7.41 (m, 7H), 3.75 (t, *J* = 6.2 Hz, 2H), 2.72 (t, *J* = 6.8 Hz, 2H), 2.19 (m, 2H); IR (cm^−1^, neat) 2924, 2227, 1743, 1587, 1543, 1359; m/z (ES Mass) 306.1 (M+1, 100%); ^13^C NMR (CDCl_3_, 200 MHz) 148.5 (2C), 142.9, 137.1, 129.7 (3C), 128.3 (3C), 126.8 (2C), 125.5 (2C), 124.1, 89.6, 81.6, 68.7, 61.9, 43.4; HRMS (ESI): calcd for C_20_H_16_ClN (M+H)^+^ 306.1050; found 306.1047.

Compound **5g**; brown solid, Rf (20% ethyl acetate/n-hexane) 0.19; ^1^H NMR (CDCl_3_, 400 MHz) δ 8.20 (d, *J* = 8.3 Hz, 1H), 7.93 (d, *J* = 8.5 Hz, 1H), 7.74 (t, *J* = 8.3 Hz, 1H), 7.66–7.64 (m, 2H), 7.58 (s, 1H), 7.53–7.37 (m, 5H), 7.12–7.0 (m, 3H), 3.7 (s, 3H); IR (cm^−1^, neat) 2929, 2214, 1589, 1288; m/z (ES Mass) 336.14 (M+1, 100%); ^13^C NMR (CDCl_3_, 200 MHz) 160.7, 158.0, 148.2 (2C), 142.7, 138.3, 132.1 (2C), 130.0 (2C), 129.5 (3C), 128.5 (2C), 127.1 (2C), 125.7 (2C), 124.2, 121.7, 90.7, 88.9, 55.2; HRMS (ESI): calcd for C_24_H_17_NO (M+H)^+^ 336.1388; found 336.1400.

Compound **5h**; low melting solid, Rf (20% ethyl acetate/n-hexane) 0.2; ^1^H NMR (CDCl_3_, 400 MHz) δ 8.21 (d, *J* = 8.1 Hz, 1H), 7.86 (d, *J* = 8.2 Hz, 1H), 7.77 (t, *J* = 6.8 Hz, 1H), 7.66–7.64 (m, 2H), 7.55–7.48 (m, 3H), 7.40–7.36 (m, 3H), 7.32–7.22 (m, 3H); IR (cm^−1^, neat) 2924, 2214, 1581, 1494; m/z (ES Mass) 324.2 (M+1, 100%); ^13^C NMR (CDCl_3_, 200 MHz) 160.4, 148.5, 147.7, 143.0, 139.6, 133.2 (2C), 132.1, 131.2 (2C), 130.5 (3C), 129.0 (3C), 128.1 (2C), 127.2, 126.5, 125.7, 90.3, 89.0; HRMS (ESI): calcd for C_23_H_14_FN (M+H)^+^ 324.1189; found 324.1195.
